# Protein target identification and toxicological mechanism investigation of silver nanoparticles-induced hepatotoxicity by integrating proteomic and metallomic strategies

**DOI:** 10.1186/s12989-019-0322-4

**Published:** 2019-11-27

**Authors:** Ming Xu, Qiuyuan Yang, Lining Xu, Ziyu Rao, Dong Cao, Ming Gao, Sijin Liu

**Affiliations:** 1 State Key Laboratory of Environmental Chemistry and Ecotoxicology, Research Center for Eco-Environmental Sciences, Chinese Academy of Sciences, Beijing, 100085 China; 2 University of Chinese Academy of Sciences, Beijing, 100049 China

**Keywords:** Silver nanoparticles, Silver ion, Hepatotoxicity, Protein target, Glutathione S-transferase

## Abstract

**Background:**

Silver nanoparticles (AgNPs), as promising anti-microbials and anti-cancer therapeutics, the toxicological effect and killing efficiency towards cells need in-depth investigation for better applications in daily life and healthcare fields. Thus far, limited studies have yet elucidated the protein targets of AgNPs and silver ions (Ag^+^) released from intracellular AgNPs dissolution in hepatocytes, as well as potential interaction mechanism.

**Results:**

Through integrating proteomic and metallomic methodologies, six intracellular protein targets (i.e. glutathione S-transferase (GST), peroxiredoxin, myosin, elongation factor 1, 60S ribosomal protein and 40S ribosomal protein) were ultimately identified and confirmed as AgNPs- and Ag^+^ −binding proteins. Toward a deep understanding the direct interaction mechanism between AgNPs and these protein targets, GST was chosen as a representative for toxicological investigation. The results revealed that AgNPs could remarkably deplete the enzyme activity of GST but did not depress the expressions, resulting in elevated intracellular oxidative stress and cell death. Finally, both “Ag^+^ effect” and “particle-specific effect” were demonstrated to concomitantly account for the overall cytotoxicity of AgNPs, and the former relatively contributed more via activity depletion of GST.

**Conclusions:**

Collectively, our major contribution is the development of an efficient strategy to identify the intracellular AgNPs-targeted protein (e.g. GST) through integrating proteomic and metallomic methodologies, which is helpful to accelerate the interpretation of underlying toxicological mechanism of AgNPs.

## Background

As a new generation of anti-microbials, silver nanoparticles (AgNPs) have been extensively applied in commercial goods as additive, due to the excellent broad-spectrum antimicrobial activity [1, 2]. Besides, AgNPs are also regarded as potential tools for biomedical applications such as biosensing, anti-viral, anti-cancer, etc. [1, 3, 4]. However, the presence of AgNPs in toothbrush, textile, food packing and medical products raises the concerns about the human health risks [1, 5–8]. For instance, liver is well known as the primary biological barrier to remove foreign nanoparticles by cells of mononuclear phagocyte system [9]. Reported studies have already found that liver accumulated the highest level of AgNPs in animals [10–13], which could finally lead hepatotoxicity [13–15]. Although many in vitro and in vivo studies have been carried out to investigate the underlying toxicological mechanisms of AgNPs-induced hepatotoxicity, some knowledge gaps still exist which hamper the understanding of their health risks [7, 14].

Generally, the toxicity of AgNPs mainly originates from the degraded forms of AgNPs, “particle-specific effect” or the triggered oxidation stress [16]. After cellular internalization, AgNPs would enter the acidic endo/lysosomes (pH 4.5–6.5) [17], and undergo chemical transformation from particulate silver to elemental silver, Ag^+^, Ag-O- and Ag-S- species [16]. The Ag^+^ released from AgNPs dissolution prefers to bind intracellular sulfhydryl group (−SH)-containing molecules and leads cytotoxicity, which is known as the “Trojan-horse” mechanism [18]. On the other hand, the “particle-specific effect” of AgNPs can not be neglected because the intrinsic physicochemical properties of AgNPs (e.g. shape, size and surface modification) are critical important for their biomolecular interaction and toxicological effect [19, 20]. Besides, AgNPs can also promote intracellular reactive oxygen species (ROS) production and cause serious cellular damages, e.g. genotoxicity, mitochondrial dysfunction and cell membrane damage [18].

**Fig. 1:**
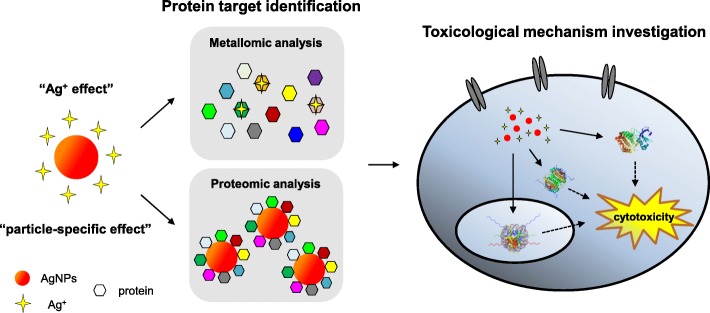
Schematic illustrations of protein target identification and toxicological mechanism investigation of AgNPs-induced cytotoxicity

In order to uncover underlying toxicological mechanisms of AgNPs, many efforts have been devoted to elaborate the impacts of AgNPs on various cellular signaling pathways and biomarker expression [18]. However, few study concerns about the intracellular protein targets which directly interact with AgNPs until now, because it is a great challenge to discriminate the key protein targets from thousands of proteins inside cell. Besides, the impact of “Ag^+^ effect” and “particle-specific effect” originated from AgNPs on protein molecules still lacks enough investigation. For the above reasons, new strategies are urgently expected to be developed to screen and identify the potential protein targets of AgNPs. Proteomic and metallomic methodologies are powerful tools for searching and discovering key protein targets of metallic nanoparticles and ions as we previously reported [21, 22]. In this study, we integrated both methodologies to screen and identify the intracellular AgNPs– and Ag^+^–binding proteins, which are responsible for the cytotoxicity of AgNPs on hepatic cells as shown in Fig. 1. Finally, the underlying toxicological mechanism was demonstrated to discriminate “Ag^+^ effect” and “particle-specific effect” of AgNPs responsible for cytotoxicity.

## Results and discussion

### Characterization of AgNPs

**Fig. 2:**
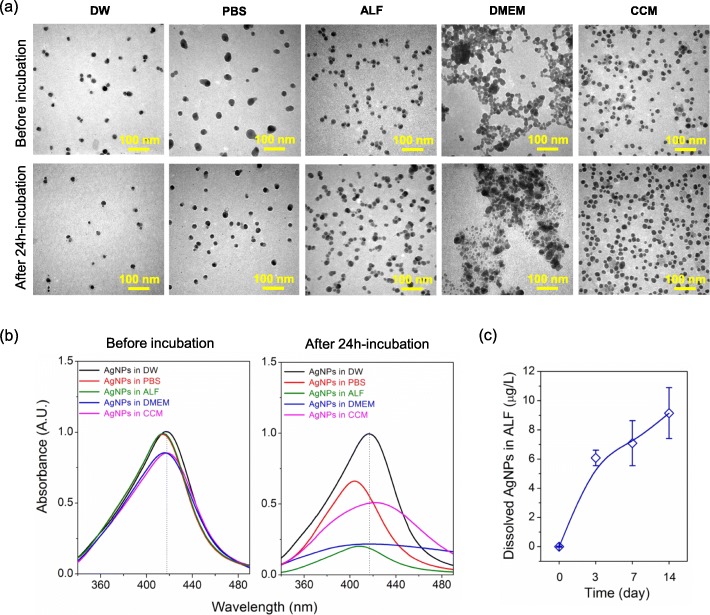
Physicochemical characterization of AgNPs. **a** Representative TEM images and (**b**) UV-visible spectra of AgNPs in DW, PBS, ALF, DMEM and CCM before or after 24 h-incubation at 37 °C. **c** The dissolution of AgNPs in ALF from 0 to 14 days

AgNPs used in this study were nanospheres with polyvinylpyrrolidone (PVP) coating, which is a typical surface modification for sterically stabilizing AgNPs in solution [23, 24]. In order to gain an insight into the cytotoxicity, the physicochemical properties of AgNPs were first characterized in various media (i.e. deionized water (DW), phosphate buffer saline (PBS), artificial lysosomal fluid (ALF), Dulbecco’s Modified Eagle Medium (DMEM) and cell culture medium (CCM)). PBS (pH 7.2–7.4) and ALF (pH 4.5–5.0) were respectively utilized to represent human body fluid and lysosomal fluid [25, 26], while DMEM and CCM (DMEM supplemented with 10% fetal bovine serum) were utilized to compare the impact of protein corona on AgNPs. As observed in Fig. 2a, AgNPs were well dispersed and relatively stable in PBS, ALF and CCM compared to pristine AgNPs within 24 h, but serious agglomeration occurred in DMEM, which is also supported by the data of hydrodynamic diameter in Table 1. Then, the morphology and diameter of AgNPs were studied by transmission electron microscopy (TEM). In Table 1, it shows that the initial diameters of AgNPs were 19.4 ± 3.5 nm, 34.6 ± 14.4 nm, 21.0 ± 5.0 nm and 19.7 ± 3.5 nm in DW, PBS, ALF and CCM, respectively. After 24 h-incubation at 37 °C, those changed to be 19.8 ± 4.3 nm, 23.5 ± 4.7 nm, 22.3 ± 5.9 nm and 19.8 ± 4.2 nm, respectively. These results indicate that AgNPs exhibited good stability in DW and CCM within 24 h, but partial aggregation, dissolution and recrystallization of AgNPs existed in PBS and ALF due to the high ionic strength or acidic pH (Additional file 1: Table S1). The above findings were further confirmed by UV–visible absorption spectroscopy in Fig. 2b, which revealed that the surface plasmon resonance peak at *λ*_max_ 417 nm respectively decreased with a blue shift to 403 nm or 408 nm for AgNPs in PBS or ALF after 24 h-incubation, suggesting the dissolution of AgNPs occurred [27]. Conversely, a red shift from 417 nm to 423 nm was observed for AgNPs in CCM, due to the protein adsorption on AgNPs [28]. Additionally, the zeta-potential of AgNPs in CCM were determined to be − 14.5 ± 0.5 mV and − 16.2 ± 0.2 mV before and after 24 h-incubation (Table 1), indicating AgNPs were negatively charged during experimental process.

**Table 1:** Physicochemical characterization of AgNPs in various media

	DW	PBS	ALF	DMEM	CCM
Diameter measured by TEM (nm) ^c^	19.4 ± 3.5 ^a^	34.6 ± 14.4 ^a^	21.0 ± 5.0 ^a^	–	19.7 ± 3.5 ^a^
	19.8 ± 4.3 ^b^	23.5 ± 4.7 ^b^	22.3 ± 5.9 ^b^	–	19.8 ± 4.2 ^b^
Hydrodynamic diameter (nm)	58.1 ± 0.3 ^a^	96.6 ± 1.6 ^a^	71.1 ± 3.1 ^a^	113.2 ± 11.6 ^a^	103.6 ± 7.1 ^a^
	60.2 ± 0.4 ^b^	67.6 ± 3.2 ^b^	96.9 ± 4.2 ^b^	402.1 ± 61.7 ^b^	82.6 ± 0.4 ^b^
Zeta-potential (mV)	−1.5 ± 0.4 ^a^	− 17.4 ± 0.3 ^a^	− 11.9 ± 1.7 ^a^	− 11.8 ± 0.8 ^a^	−14.5 ± 0.5 ^a^
	− 1.5 ± 0.6 ^b^	−27.9 ± 0.8 ^b^	−14.9 ± 1.6 ^b^	−13.0 ± 0.3 ^b^	−16.2 ± 0.2 ^b^
Polydispersity index (PDI)	0.15 ± 0.01 ^a^	0.46 ± 0.03 ^a^	0.45 ± 0.07 ^a^	0.51 ± 0.09 ^a^	0.40 ± 0.03 ^a^
	0.14 ± 0.00 ^b^	0.41 ± 0.01 ^b^	0.49 ± 0.10 ^b^	0.76 ± 0.13 ^b^	0.28 ± 0.01 ^b^
*λ*_max_ (nm)	417 ^a^	415 ^a^	415 ^a^	416 ^a^	418 ^a^
	417 ^b^	403 ^b^	408 ^b^	420 ^b^	423 ^b^

^a^t = 0 h; ^b^ t = 24 h. ^c^ AgNPs counting (*n* = 100)

Once cells were exposed to AgNPs in CCM, protein-adsorbed AgNPs will be internalized by cells and enter the acidic endo/lysosomes (pH 4.5–6.5). To uncover the possible mechanism during this process, AgNPs were first pre-incubated in CCM for 1 h at 37 °C and isolated as protein-adsorbed AgNPs. Then, the protein-adsorbed AgNPs were added into ALF for further determination (Additional file 1: Figure S1). As observed in Fig. 2c, the dissolved AgNPs in ALF increased along with incubation from 0 to 14 days, which was also confirmed by the blue shift of *λ*_max_ from 415 nm to 402 nm in this process (Additional file 1: Figure S1a). Simultaneously, the hydrodynamic diameter increased from 71.1 ± 3.1 nm to 262.7 ± 95.7 nm (Additional file 1: Figure S1b), indicating serious aggregation occurred in ALF. Collectively, the above findings demonstrated that the used AgNPs were relatively stable due to protein adsorption in CCM before cellular internalization. After the entry into acidic endo/lysosomes, AgNPs may aggregate and dissolve to Ag^+^ inside cells, which will trigger cytotoxicity.

### Cytotoxicity of AgNPs on hepatic cells

**Fig. 3:**
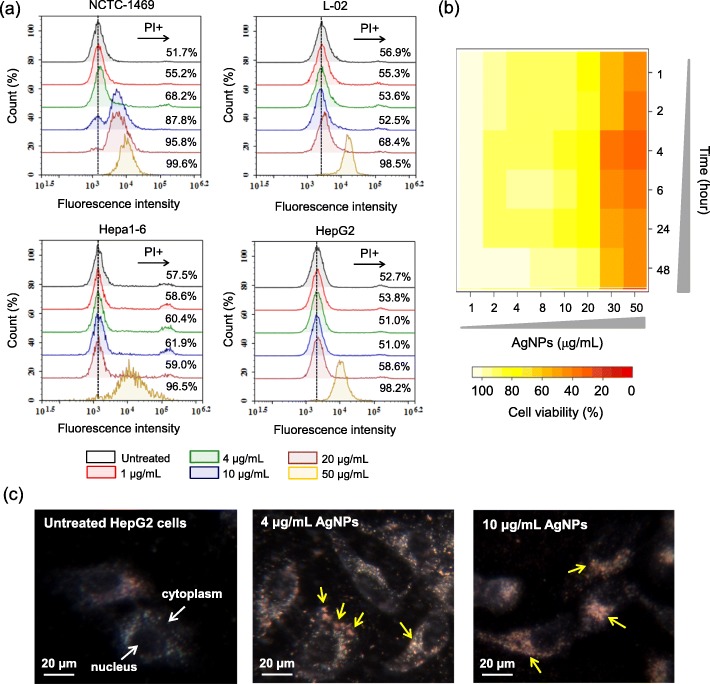
Cytotoxicity of AgNPs. **a** Cell death of NCTC-1469, L-02, Hepa1–6 and HepG2 cells with or without AgNPs-treatment at 1, 4, 10, 20 or 50 *μ*g/mL analyzed using flow cytometer. **b** Heatmap of cell viability. HepG2 cells were treated with AgNPs at 1, 2, 4, 8, 10, 20, 30 or 50 *μ*g/mL for 1, 2, 4, 6, 24 and 48 h, respectively. **c** Hyperspectral images of untreated and AgNPs-treated HepG2 cells at 4 or 10 *μ*g/mL for 24 h. The yellow arrow denotes the internalized AgNPs in cytoplasm

Liver is the predominant organ of in vivo AgNPs accumulation as reported [12, 14, 15, 29], which could induce serious hepatotoxicity [14, 15]. On the basis of above findings, two normal hepatic cell lines (NCTC-1469 and L-02) and two hepatoma cell lines (Hepa1–6 and HepG2) were first used to investigate the cytotoxicity of AgNPs. For these hepatic cells, their viability started to be significantly inhibited when the concentration of AgNPs reached 10 *μ*g/mL (Additional file 1: Figure S2). Following, cell death induced by AgNPs was assessed based on propidium iodide (PI) staining assay. As observed in Fig. 3a, after 24 h AgNPs-treatment over 10 *μ*g/mL, the proportion of PI positive cells (i.e. dead cells) all increased for both normal hepatic cells (i.e. NCTC-1469 and L-02) and hepatoma cells (i.e. Hepa1–6 and HepG2), which is basically identical to the results of cell viability (Additional file 1: Figure S2). Then, HepG2 cells were chosen in following studies to investigate the potential toxicological mechanism. The cytotoxicity of AgNPs on HepG2 cells was carefully examined at various exposure doses (1, 2, 4, 8, 10, 20, 30 and 50 *μ*g/mL) and time points (1, 2, 4, 6, 24 and 48 h). In Fig. 3b, it shows that the cytotoxicity of AgNPs on HepG2 cells mainly manifests dose-response manner rather than time-response manner. At all time points, the viability of AgNPs-treated HepG2 cells was not significantly affected less than 10 *μ*g/mL, thus the following mechanism investigations were mainly carried out below this sublethal concentration (i.e. 10 *μ*g/mL).

### Intracellular interaction between AgNPs and proteins

**Fig. 4:**
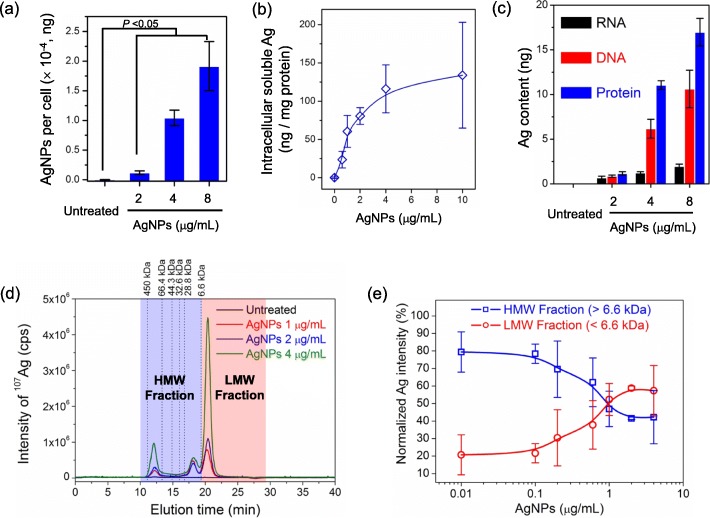
Internalization of AgNPs and intracellular soluble Ag-components in AgNPs-treated HepG2 cells. **a** Cellular delivered dose of AgNPs at 2, 4 or 8 *μ*g/mL after 24 h-treatment (*n* = 3). **b** Soluble Ag-components of HepG2 cells treated with AgNPs at 0.6, 1, 2, 4 or 10 *μ*g/mL for 24 h (*n* = 3). **c** Ag content of RNA, DNA and protein components in AgNPs-treated HepG2 cells at 2, 4 or 8 *μ*g/mL for 24 h (*n* = 5). **d** Chromatograms of Ag-protein complexes from untreated and AgNPs-treated HepG2 cells at 1, 2 or 4 *μ*g/mL for 24 h analyzed using SEC-ICP-MS. **e** Normalized HMW and LMW fractions from AgNPs-treated HepG2 cells at 0.01, 0.1, 0.2, 0.6, 1, 2 or 4 *μ*g/mL for 24 h analyzed using SEC-ICP-MS (*n* = 3)

As described above, once AgNPs enter cells through internalization, dissolution will occur in acidic endo/lysosomes. To investigate possible interaction between AgNPs and proteins, the cellular internalization and location of AgNPs were first examined using hyperspectral microscopy (Fig. 3c). After 24 h-treatment at 4 or 10 *μ*g/mL, AgNPs were mainly observed in endo/lysosomes as reported [30]. Besides in situ analysis, the cellular internalization of AgNPs was quantitatively analyzed using inductively coupled plasma mass spectrometry (ICP-MS). In Fig. 4a, it shows that the internalization of AgNPs was a positive relationship to its exposure concentration below 10 *μ*g/mL. At the same time, the intracellular soluble Ag-components were collected and quantitatively analyzed through cell lysis and centrifugation to remove AgNPs and insoluble Ag-component (Fig. 4b and Additional file 1: Figure S3). The results reveal that the intracellular soluble Ag-components increased along with internalized AgNPs, which means internalized AgNPs continue to transform to the soluble species during dissolution process. For instance, Ag^+^ released from intracellular AgNPs dissolution can form soluble Ag-complexes with biomacromolecules. Consequently, the components of RNA, DNA and protein in AgNPs-treated HepG2 cells were simultaneously isolated as reported [31], and their Ag contents were determined (Fig. 4c). Obviously, protein molecules bonded more Ag, which is 0.79 and 7.95 times higher than DNA and RNA after AgNPs-treatment at 4 *μ*g/mL, respectively.

Furthermore, the soluble Ag-protein complexes were analyzed using size-exclusion chromatography coupled ICP-MS (SEC-ICP-MS), which is a classic technique to characterize metalloprotein. In Fig. 4d, the Ag-protein complexes can be classified into high molecular weight (HMW, > 6.6 kDa) and low molecular weight (LMW, < 6.6 kDa) fractions, because metallothionein (ca. 6.6 kDa) is generally regarded as LMW protein [32]. Besides, the LMW fraction also contained small biomolecules such as glutathione (GSH). According to Ag signals in Fig. 4d, three major chromatographic peaks can be discriminated, which were eluted at 12.0 min, 18.1 min and 20.3 min. After normalization of these chromatographic peaks, the relative changes of HMW and LMW fractions can be compared (Fig. 4e), which reveals that the relative proportion of HMW fraction continued to decrease along with increasing concentration of AgNPs from 0.01 to 4 *μ*g/mL. This phenomenon suggests that the binding of HMW proteins to Ag^+^ became saturation and more LMW molecules (e.g. metallothionein and GSH) were continually synthesized by cells to neutralize AgNPs and Ag^+^ for detoxification. These findings indicate that the dissolution of internalized AgNPs resulted the formation of Ag-protein complexes. As a consequence, the damage of protein molecules by AgNPs and Ag^+^ would destroy intracellular homeostasis.

### Protein target identification based on proteomic and metallomic strategies

**Fig. 5:**
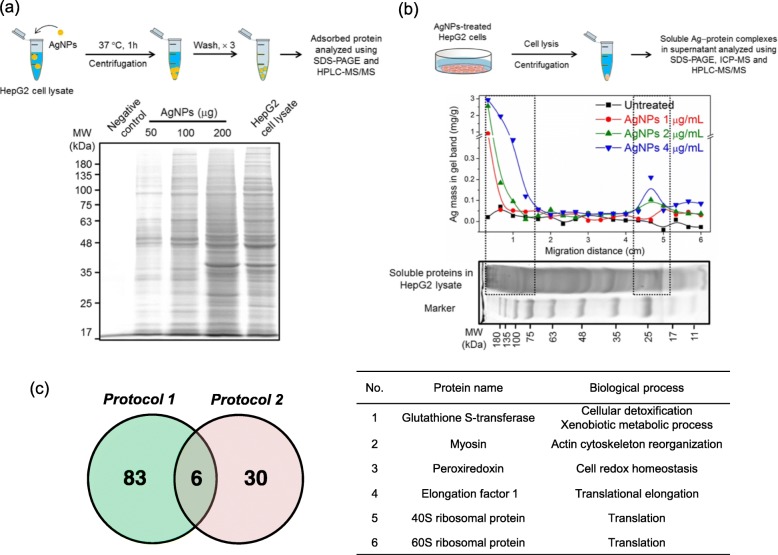
Identification of AgNPs– and Ag^+^–binding proteins based on proteomic and metallomic strategies. **a** A schematic showing “protocol 1” for proteomic analysis of protein adsorbed on AgNPs. Experimental procedure is presented in the upper panel. Briefly, a does of 50, 100 or 200 *μ*g AgNPs was added into HepG2 cell lysate for 1 h incubation at 37 °C, and adsorbed proteins on AgNPs were isolated and identified using SDS-PAGE and HPLC-MS/MS. **b** A schematic showing “protocol 2” for metallomic analysis of Ag-protein complexes in AgNPs-treated HepG2 cells at 1, 2 or 4 *μ*g/mL for 24 h. Experimental procedure is presented in the upper panel. Briefly, untreated or AgNPs-treated HepG2 cells were harvested for cell lysis. Then, the soluble Ag-protein complexes in cell lysate were separated and analyzed using SDS-PAGE and ICP-MS. Finally, the detected Ag^+^-binding proteins were identified using HPLC-MS/MS. **c** Identification of protein targets by both “protocol 1” and “protocol 2” depicted as a Venn diagram. The detailed information of these proteins was provided in Additional file 1: Table S2 and S3. The involved biological process of proteins is obtained from UniProt (https://www.uniprot.org/)

Inside cells, AgNPs can quickly absorb surrounding proteins and affect their structure and function [33–35]. On the other hand, the intracellular dissolution of AgNPs can release Ag^+^ to damage proteins as mentioned above. Hence, we study the intracellular protein targets of AgNPs from these two aspects, which are believed to play important roles in cytotoxicity of AgNPs. First, the adsorbed proteins on AgNPs in HepG2 cell lysate were analyzed based on proteomic strategy, and the experimental process is illustrated as “protocol 1” in Fig. 5a. Briefly, the adsorbed proteins on AgNPs in cell lysates were isolated by centrifugation and sodium dodecyl sulfate-polyacrylamide gel electrophoresis (SDS-PAGE), and identified by high-performance liquid chromatography-tandem mass spectrometry (HPLC-MS/MS). Notably, the pattern of gel lanes varied with the amount of AgNPs from 50 to 200 *μ*g in Fig. 5a, which were markedly different from that of HepG2 cell lysate. This phenomenon indicates that intracellular proteins exhibited diverse affinity to AgNPs. Through analysis of these characteristic protein bands, a total of 89 proteins were identified as major compositions of adsorbed proteins (i.e. protein corona) on AgNPs in Additional file 1: Table S2. Some of these proteins are closely correlated with the cellular processes of cytoskeleton assembly (e.g. myosin, tubulin and actin), glycolysis (e.g. pyruvate kinase and glyceraldehyde-3-phosphate dehydrogenase), translation (e.g. ribosomal proteins) and catalytic cycle (e.g. cytochrome P450), which have been reported to be affected by AgNPs [36–39]. It has to be underlined that these identified proteins are relatively high abundant components, and more low abundant proteins may not be detected due to the limitation of analytical technique. Still, the proteins in Additional file 1: Table S2 are regarded as important candidates to directly interact with AgNPs.

On the other side, to reveal the protein targets of Ag^+^ released from intracellular AgNPs dissolution, metallomic strategy was utilized to screen and identify the “Ag-protein complexes”, and the experimental process is illustrated as “protocol 2” in Fig. 5b. Briefly, after 24 h AgNPs-treatment at 1, 2 or 4 *μ*g/mL, HepG2 cells were harvested. After cell lysis, the soluble proteins were isolated and separated using SDS-PAGE as shown in Fig. 5b. Then, the gels were cut into aliquots, acid digested and analyzed for screening “Ag-protein complexes” by ICP-MS. In Fig. 5b, two obvious Ag signals in gels can be respectively observed at MW > 75 kDa and 30 kDa > MW > 17 kDa, which are in good agreement with the results in Fig. 4d. As we could not exclude the presence of other HMW Ag-containing components (e.g. DNA) at MW > 75 kDa, further analysis of this fraction in gels was abandoned. Meanwhile, the proteins at 30 kDa > MW > 17 kDa were analyzed using HPLC-MS/MS, and 36 proteins were identified in Additional file 1: Table S3. Then, these proteins were matched with those in Additional file 1: Table S2 for further confirmation, and six of them were ultimately identified as vital protein targets of AgNPs as shown in Fig. 5c, including glutathione S-transferase, myosin, peroxiredoxin, elongation factor 1, 60S ribosomal protein and 40S ribosomal protein. Among them, glutathione S-transferase, myosin and peroxiredoxin contain free –SH, which can serve as strong evidence of AgNPs– or Ag^+^–binding site [40–42].

### AgNPs-induced cytotoxicity through depleting GST activity

On the basis of above findings, GST was chosen as a representative protein target of AgNPs to investigate the toxicological mechanism, which is known as an important detoxification enzyme [43]. Glutathione S-transferases (GSTs) locate in cytosol, mitochondria and microsome, comprising a large family of key defense enzyme against xenobiotic toxicity through catalyzing the conjugation of GSH to substrates [42]. GSTs can constitute up to 10% of cytosolic protein in some mammalian organs [44]. On the other hand, GSTs are destined as key biomarkers for cancer because of their critical roles in carcinogenesis and chemotherapeutic drug resistance [45]. For instance, high expression of GSTs in cancerous cells is positively correlated with cisplatin resistance of tumors, and inhibition of their activity can increase antitumor efficiency of drugs [46]. Therefore, it is reasonable to study the interaction between AgNPs and GSTs from both toxicological and medical aspects.

**Fig. 6:**
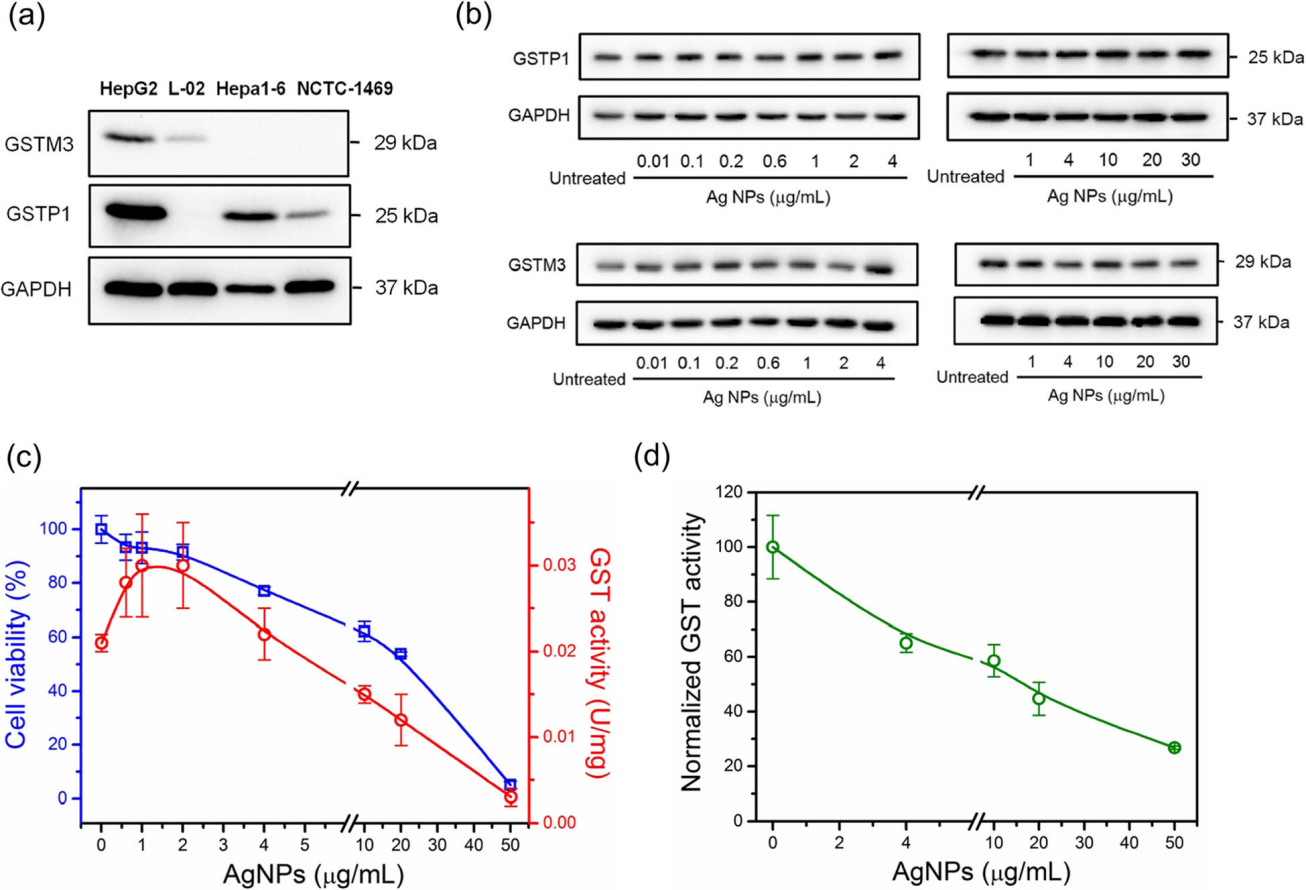
Impact of AgNPs on the expression and enzyme activity of GSTs. **a** Western blot analysis of GSTP1 and GSTM3 in four hepatic cell lines (NCTC-1469, L-02, Hepa1–6 and HepG2). **b** Western blot analysis of GSTP1 and GSTM3 in untreated and AgNPs-treated HepG2 cells at 0.01, 0.1, 0.2, 0.6, 1, 2, 4, 10, 20 or 30 *μ*g/mL for 24 h. **c** Cell viability and intracellular GST enzyme activity of untreated and AgNPs-treated HepG2 cells at 0.6, 1, 2, 4, 10, 20 or 50 *μ*g/mL for 24 h (*n* = 5). **d** Depletion of GST enzyme activity by AgNPs under cell-free conditions (*n* = 3)

In Additional file 1: Table S2 and S3, two GST isoenzymes (i.e. glutathione S-transferase P1 (GSTP1) and glutathione S-transferase Mu 3 (GSTM3)) were identified as intracellular protein targets of AgNPs, which are high abundant in liver [47]. So the level of GSTP1 and GSTM3 in four hepatic cell lines (NCTC-1469, L-02, Hepa1–6 and HepG2) was first compared by Western Blot. As observed in Fig. 6a, the level of GSTP1 in Hepa1–6 and HepG2 cells is much higher. For GSTM3, its level is higher in HepG2 cells, but relatively low in L-02, Hepa1–6 and NCTC-1469 cells. These results illustrate that GSTs indeed highly expressed in hepatoma cells as reported [48], which is benefit to study the interaction between GSTs and AgNPs.

**Fig. 7:**
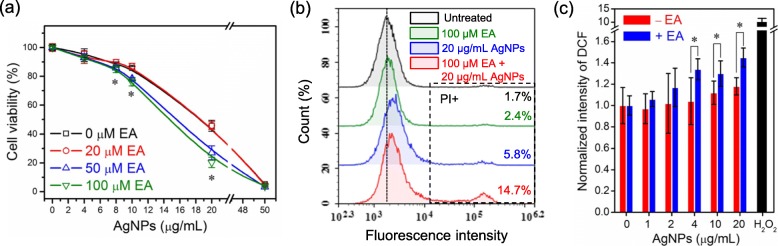
Depletion of GST activity induced more serious cytotoxicity. **a** Cell viability of untreated and AgNPs-treated HepG2 cells at 4, 8, 10, 20 or 50 *μ*g/mL for 24 h without or with EA-treatment at 20, 50 or 100 *μ*M (*n* = 5). **b** Cell death of untreated and AgNPs-treated HepG2 cells at 20 *μ*g/mL for 24 h without or with EA-treatment at 100 *μ*M. **c** Intracellular ROS generation in untreated and AgNPs-treated HepG2 cells at 1, 2, 4, 10 or 20 *μ*g/mL for 3 h without or with EA-treatment at 100 *μ*M (n = 5). H_2_O_2_-treatment was used as positive control. Asterisk (*) denotes *P* < 0.05

After 24 h AgNPs-treatment at 0.01, 0.1, 0.2, 0.6, 1, 2, 4, 10, 20 or 30 *μ*g/mL, the expressions of GSTP1 and GSTM3 in HepG2 cells were assessed by Western Blot. As observed in Fig. 6b, the intracellular levels of GSTP1 and GSTM3 retained stable compared with untreated cells even if AgNPs reached 30 *μ*g/mL, which is a high dose inducing cytotoxicity. However, the enzyme activity assay of GSTs revealed that the GST activity significantly changed after AgNPs-treatment (Fig. 6c). When HepG2 cells were treated with low-dose AgNPs, intracellular GST activity increased to 1.33, 1.43 and 1.43 times compared to that of untreated cells at 0.6, 1 and 2 *μ*g/mL, respectively. Conversely, it decreased to 0.71, 0.57 and 0.14 times after AgNPs-treatment at 10, 20 and 50 *μ*g/mL, respectively. These changes imply that intracellular GSTs could be activated to protect cells by low-dose AgNPs, but their activity was depleted by high-dose AgNPs. In addition to cellular experiments, a commercial GST purified from equine liver was used to confirm its interaction with AgNPs under cell-free conditions. The results show that AgNPs could directly deplete 34.9, 41.4, 55.3 and 73.2% of GST activity at 4, 10, 20 and 50 *μ*g/mL, respectively. For further validation, ethacrynic acid (EA) was used to suppress GST activity as an inhibitor [46]. In Additional file 1: Figure S4a, the experimental results revealed that EA could inhibit 35.7 and 43.8% of GST activity at 50 μM and 100 *μ*M, respectively. Simultaneously, this GST suppression led the decreasing cell viability and increasing intracellular ROS (Additional file 1: Figure S4b and c). Additionally, the cytotoxicity of AgNPs was investigated with or without EA-treatment in the aspects of cell viability, cell death and ROS generation. In Fig. 7, it shows that cell viability, cell death and intracellular ROS generation respectively changed from 45.6 to 20.3%, 5.8 to 14.7% and 1.18-fold to 1.45-fold without or with EA-treatment at 100 *μ*M, when HepG2 cells were treated with AgNPs at 20 *μ*g/mL. The aggravated cytotoxicity indicates that the presence of EA sensitized HepG2 cells to AgNPs, proving the importance of GST detoxification and the interaction between GSTs with AgNPs. All of above results illustrate that AgNPs could indeed lead intracellular oxidation stress and cytotoxicity through acting GST molecules and suppressing its enzyme activity, although GST expressions were not significantly affected.

### “Ag^+^ effect” and “particle-specific effect” on depleting GST activity

**Fig. 8:**
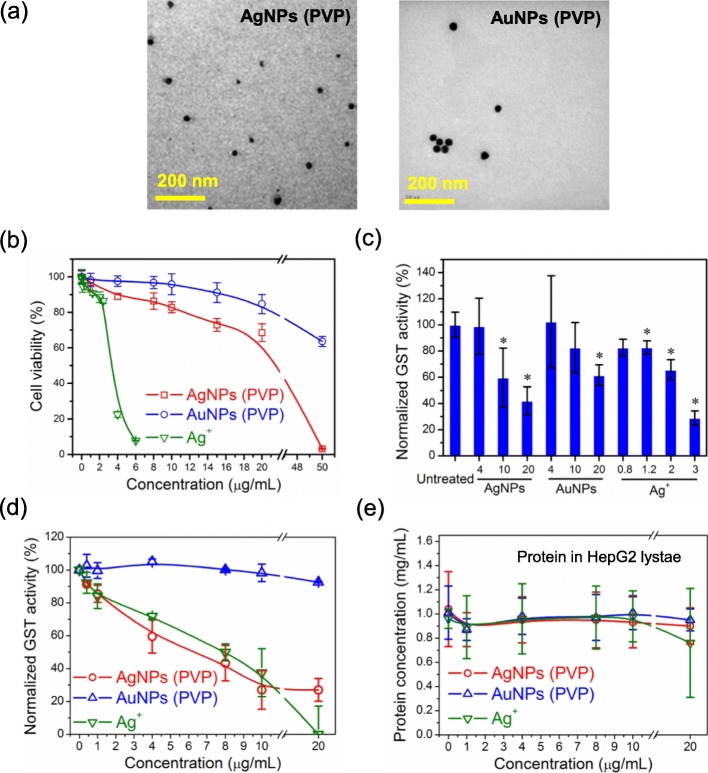
Influence of AgNPs, AuNPs and Ag^+^ on cytotoxicity and GST activity. **a** Representative TEM images of AgNPs and AuNPs. **b** Cell viability of HepG2 cells treated with AgNPs or AuNPs at 1, 4, 8, 10, 15, 20 or 50 *μ*g/mL for 24 h, or Ag^+^ at 0.04, 0.1, 0.4, 1.2, 2, 2.4 or 4 *μ*g/mL for 24 h (*n* = 5). **c** Normalized intracellular GST enzyme activity of HepG2 cells treated with AgNPs or AuNPs at 4, 10 or 20 *μ*g/mL for 24 h, or Ag^+^ at 0.8, 1.2, 2 or 3 *μ*g/mL for 24 h (*n* = 4). **d** Normalized GST enzyme activity and (e) protein concentration in cell lysates after incubation for 1 h at 37 °C with AgNPs, AuNPs or Ag^+^ at 0.4, 1, 4, 8, 10 or 20 *μ*g/mL (*n* = 3). Asterisk (*) denotes *P* < 0.05, compared to untreated cells

In order to investigate the “Ag^+^ effect” and “particle-specific effect” from AgNPs, the impact of AgNPs on GST was compared with Ag^+^ and gold nanoparticles (AuNPs). The used AuNPs were PVP-coated nanosphere with comparable hydrodynamic diameter (57.5 ± 0.2 nm) to AgNPs as shown in Fig. 8a. Because AuNPs nearly do not release gold ion, it can partially reflect the “particle-specific effect” of AgNPs on the basis of similar physicochemical properties inside cells (Additional file 1: Figure S5). In Fig. 8b, the impact of AgNPs, AuNPs and Ag^+^ on cell viability of HepG2 was first compared, which exhibited a dose-response manner of decrease in the sequence of Ag^+^, AgNPs and AuNPs. The cytotoxicity of Ag^+^ (IC50 = 3 *μ*g/mL) was almost one order of magnitude higher than AgNPs (IC50 = 30 *μ*g/mL). Although AuNPs are generally considered to be biocompatible and less toxic, a significant decrease of 36.4% cell viability occurred at a high dose of 50 *μ*g/mL, which was probably induced by negative charge or PVP ligands on AuNPs (Additional file 1: Figure S6). In Fig. 8c, it shows that the depletion of intracellular GST activity by AgNPs, AuNPs and Ag^+^ was in accordance with the tendency of cell viability. After AgNPs-, AuNPs- and Ag^+^-treatment at 20 *μ*g/mL or 2 *μ*g/mL, intracellular GST activity remained 42, 61 and 66%, respectively. Additionally, AgNPs, AuNPs and Ag^+^ were directly added into isolated HepG2 cell lysate, respectively. After 1 h incubation at 37 °C, GST activity was determined as shown in Fig. 8d and e. The results reveal that both AgNPs and Ag^+^ could quickly deplete GST activity compared to AuNPs without the denaturation of protein in cell lysates. These findings provide more solid evidence for the “Ag^+^ effect” and “particle-specific effect” from AgNPs at molecular level. Although “Ag^+^ effect” shows the predominant contribution of cytotoxicity from AgNPs, the “particle-specific effect” can also not be overlooked as observed in Fig. 8b, c and d. Discarding ion release, the cellular internalization, intracellular degradation and biomolecular interaction of AuNPs may bring hazards to cells, such as ligand release, protein destruction, oxidative stress, etc. [19].

## Conclusion

In conclusion, the intracellular protein targets and toxicological mechanisms of AgNPs were carefully examined respect to human health risks. Six vital protein targets (i.e. glutathione S-transferase, myosin, peroxiredoxin, elongation factor 1, 60S ribosomal protein and 40S ribosomal protein) of AgNPs were ultimately identified by integrating proteomic and metallomic strategies, and the depletion of GST activity by AgNPs was revealed as a key mechanism leading cytotoxicity. At last, our results demonstrated that “Ag^+^ effect” and “particle-specific effect” originated from AgNPs differentially contributed to the overall cytotoxicity on hepatic cells. Yet, some limitations and problems are urged to be solved in future. For example, although proteomic and metallomic strategies allow us to get a bunch of protein targets of AgNPs, it is still not sufficient to reveal their interactions with AgNPs and importance on cytotoxicity. Moreover, the physicochemical properties can remarkably affect the interaction between protein targets and AgNPs, e.g. surface coating. And it is necessary to carry out more investigations with diverse surface modification except PVP-coating. We believe that further studies will greatly help us to better understand the health risks of AgNPs and obtain safer and more efficient AgNPs for practical applications.

## Methods

### Characterization of AgNPs and AuNPs

The PVP-coated AgNPs was a commercial product, and purchased from Shanghai Huzheng Nanotechnology Co., Ltd., China. The PVP-coated AuNPs were synthesized as we previously described [21]. The zeta (*ζ*)-potential, hydrodynamic diameter and PDI of AgNPs and AuNPs were assessed using a Zetasizer (Malvern Nano series, Malvern, U.K.). The UV-visible absorption spectra was measured using a UV-2800 UV/Vis spectrophotometer (Unico Instrument Co., Ltd., Shanghai, China). TEM analysis of AgNPs and AuNPs was performed on a H-7500 transmission electron microscope (Hitachi Scientific Instruments, Japan).

To study the stability and dissolution process, AgNPs were added into DW, PBS, ALF, DMEM or CCM to a final concentration of 10 *μ*g/mL and incubated at 37 °C for 24 h. For the measurement of zeta (*ζ*)-potential, hydrodynamic diameter and PDI in PBS, ALF, DMEM or CCM, AgNPs were isolated by centrifugation after incubation and redissolved in DW to get rid of the interference from the compositions in these media, such as salt and protein. For TEM measurement, the same procedure was followed, and the samples were prepared by depositing a small drop of solution onto a carbon-coated copper electron microscopy grid (Beijing Zhongjingkeyi Technology Co., Ltd., China) and then dried under room temperature. To simulate the dissolution process in acidic endo/lysosomes, AgNPs were first pre-incubated in CCM for 1 h at 37 °C and purified as protein-adsorbed AgNPs. Then, the protein-adsorbed AgNPs were added into ALF and incubated at 37 °C for 1, 3, 7 and 14 days as shown in Additional file 1: Figure S1.

### Cell culture and cytotoxicity assay

Murine hepatic cell line NCTC-1469, human hepatic cell line L-02, mouse hepatoma cell line Hepa1–6 and human hepatoma cell line HepG2 were obtained from the Shanghai Cell Bank of Type Culture Collection of China or ATCC (USA). All cells used in this study were between passages 30 and 100, and mycoplasma-free as judged by a Mycoplasma detection kit (Solarbio Science & Technology Co., Ltd., Beijing, China). Cells were cultured in DMEM (Gibco BRL Life Technologies Inc., USA) supplemented with 10% mycoplasma-free, heat-inactivated fetal bovine serum (FBS) (Gibco, 10099–141), and 100 units/mL penicillin/streptomycin (Invitrogen) in a humidified 5% CO_2_-balanced Thermo Forma 311 incubator at 37 °C. For all experiments, 1.0 × 10^4^, 5.0 × 10^5^ or 1.0 × 10^6^ cells/well were seeded with 0.1, 1 or 2 mL cell culture medium in 96-, 12- or 6-well plates from Costar (Corning, USA). The density, morphology and growth of cells were observed using an OLYMPUS IX73 microscopy equipped with ORCA-Flash4.0 LT camera (Hamamatsu Photonics, Japan).

For cytotoxicity assay, cell viability was determined through alamar-blue assay. Briefly, 1.0 × 10^4^ HepG2 cells/well were seeded in 96-well plates, and cells were then treated with AgNPs, AuNPs or Ag^+^ for 24 h. After treatment, resazurin solution (Sigma-Aldrich, USA) was added into complete cell culture medium at a final concentration of 12.5 *μ*mol/L, and cells were cultured for an additional 3 h. The fluorescence was measured at an excitation wavelength of 530 nm with an emission wavelength of 590 nm using a Varioskan Flash Multimode plate reader (Thermo Fisher Scientific Inc., USA). The possible signal inference from AgNPs on alamar-blue assay was excluded by carry out a negative control.

### Hyperspectral microscopy

For in situ analysis of cellular localization of AgNPs and AuNPs, hyperspectral imaging was carried out using a CytoViva Hyperspectral Imaging System (CytoViva, Inc., Auburn, AL). After 24 h treatment, cells were fixed with 4% paraformaldehyde in PBS for 10 min, and then imaged with hyperspectral microscopy system.

### Flow cytometry

For cell death assay, 5.0 × 10^5^ NCTC-1469, L-02, Hepa1–6 or HepG2 cells/well were seeded in 12-well plates, and treated with AgNPs for 24 h. After treatment, cells were harvested by centrifugation at 1000 rpm and repeatedly washed with PBS for 3 times. Afterwards, the harvested cells were stained with 5 *μ*L PI solutions for 15 min at room temperature in dark following the manufacturer’s instructions (BD Biosciences). After staining within 1 h, 2.0 × 10^4^ cells were subjected to analysis using a NovoCyte D1040 flow cytometry (ACEA Biosciences Inc., China).

### ICP-MS analysis

To determine Ag mass, ICP-MS analysis was performed using an Agilent 7500 ICP MS instrument (Agilent, Tokyo, Japan). Briefly, 5.0 × 10^5^ or 1.0 × 10^6^ HepG2 cells/well were seeded in 12- or 6-well plates, and treated with AgNPs for 24 h. After treatment, cells were washed with PBS for 3 times, and harvested by centrifugation at 1000 rpm. For total Ag measurement, cell pellets were directly digested with concentrated HNO_3_ (GR, 65.0%, Millipore, USA) and H_2_O_2_ (GR, 30%, Sinopharm Chemical Reagent Co., Ltd., China) with a volume ratio of 3:1 at 70 °C for 6 h using a Digital Dry Block Heater (Thermo Fisher Scientific Inc., USA). For soluble Ag-components analysis, cells were first lysed in RIPA lysis buffer for 1 h at 4 °C (Solarbio Science & Technology Co., Ltd., Beijing, China). After centrifugation at 12,000 g for 10 min at 4 °C, the supernatant was collected and digested as described above. The RNA, DNA and protein components in AgNPs-treated HepG2 cells were simultaneously isolated as reported [31], and also digested as described above. All acidic digested samples were cooled down to room temperature, and diluted with deionized H_2_O to reach a final concentration of 2% HNO_3_ before ICP-MS analysis.

For SEC-ICP-MS analysis, an Agilent 1200 HPLC system (Agilent, Wilmington, DE) was coupled to the Agilent 7500 ICP-MS. A Superdex 75 10/300 GL column (300 × 10 mm I.D., 13 *μ*m, GE Healthcare, Uppsala, Sweden) was used for sample analysis. The column was calibrated with protein standards (ferritin 450 kDa, bovine serum albumin 66.4 kDa, ovalbumin 44.3 kDa, superoxide dismutase 32.6 kDa, carbonic anhydrase 28.8 kDa and metallothionein 6.6 kDa). The cell lysates were fractionated by continuous elution at 0.7 mL/min of 100 mM CH_3_COONH_4_ (pH 7.4). The isotope of ^107^Ag was monitored by ICP-MS.

### SDS-PAGE

For adsorbed protein analysis, 50, 100 or 200 *μ*g AgNPs were respectively added into HepG2 cell lysates (1 mg/mL protein) and incubated at 37 °C for 1 h to allow the adsorption of proteins on AgNPs. Then, AgNPs were spun down at 12,000 g for 30 min, and the supernatants were carefully aspirated. AgNPs pellets were repeatedly washed and centrifuged to remove excess proteins. As a negative control, an equal volume of HepG2 cell lysate without AgNPs was applied in parallel. Finally, the adsorbed proteins on AgNPs was stripped in 30 *μ*L SDS-PAGE sample buffer (200 mM Tris buffer (pH 6.8), 2% SDS, 25% glycerol, 15 mM *β*-mercaptoethanol, and 0.1% bromophenol Blue), separated by SDS-PAGE (10% separating gel and 4% stacking gel) and visualized by coomassie brilliant blue staining method with a commercial kit (Solarbio Science & Technology Co., Ltd., Beijing, China). The gel electrophoresis was performed on a MiniPROTEAN3 system (Bio-Rad Inc., CA, USA). Protein bands in gels were imaged using a Bio-Rad ChemiDoc XRS system (Bio-Rad Inc., CA, USA).

To screen soluble Ag-protein complexes, the lysates of untreated or AgNPs-treated HepG2 cells were centrifuged at 12,000 g for 30 min at 4 °C, and the supernatants were collected for SDS-PAGE. After separation by SDS-PAGE, the gel lanes were cut into 18 equal pieces, which were further acidic digested and analyzed by ICP-MS as described above.

### Protein identification by mass spectrometry

For protein identification, the protein bands in SDS-PAGE gels were collected, destained, in-gel digested and analyzed by HPLC-MS/MS under the same experimental conditions as described previously [35].

### Western blotting

After washing with PBS, harvested cells were lysed in RIPA lysis buffer supplemented with protease inhibitor cocktail (Roche, Switzerland). Protein concentrations were determined with BCA Protein Assay Kit (Solarbio Science & Technology Co., Ltd., Beijing, China). Equal amounts of proteins were subjected to SDS-PAGE and Western blotting. The primary Abs were as follows, anti-GSTM3 Ab (1:500, Proteintech, USA), anti-GSTP1 Ab (1:500, Proteintech, USA) and anti-GAPDH Ab (1:2000, Proteintech, USA). The second Abs were as follows, goat anti-mouse HRP conjugated IgG (1:4000, Proteintech, USA) and goat anti-rabbit HRP conjugated IgG (1:4000, Proteintech, USA). Western blotting signals were detected and developed using a BIO-RAD ChemiDoc XRS chemiluminescence system (Bio-Rad Inc., CA, USA).

### GST activity assay

After AgNPs-, AuNPs- or Ag^+^-treatment, cells were washed with PBS for 3 times, and harvested by centrifugation at 1000 rpm. Then, cell pellets were resuspended in cold buffer of a Glutathione S- transferase Assay Kit (Solarbio Science & Technology Co., Ltd., Beijing, China) and treated with a ultrasonic homogenizer (JY92-IIN, Ningbo Scientz Biotechnology Co.,Ltd. China) for 3 min in ice bath. After centrifugation at 12,000 g for 10 min at 4 °C, the supernatant was used for enzyme activity measurement according to the manufacturer’s instructions. For GST activity inhibition, ethacrynic acid (≥97%, Sigma-Aldrich, USA) was dissolved in dimethyl sulfoxide as the stock solution of 10 mg/mL. A commercial GST purified from equine liver (≥ 25 units/mg protein, Sigma-Aldrich, USA) was dissolved in PBS as the stock solution of 5 mg/mL.

### Intracellular ROS assay

1.0 × 10^4^ HepG2 cells/well were seeded in 96-well plates and treated with EA or AgNPs for 1 or 3 h. After treatment, 2′,7′-dichlorofluorescin diacetate (DCFH-DA, ≥97%, Sigma-Aldrich, USA) was added at a final concentration of 10 *μ*M and incubated in dark for 30 min at 37 °C before measurements. Then, cells were washed with PBS for 3 times, and the DCF fluorescence was measured using a Varioskan Flash Multimode plate reader (Thermo Fisher Scientific Inc., USA). The excitation and emission wavelength were 488 and 525 nm, respectively. The treatment with 0.05% H_2_O_2_ for 0.5 h was used as a positive control.

### Statistical analysis

The difference of the single treated group relative to untreated control was determined using independent *t-*test. The significance of mean difference for two or more treated groups relative to untreated group was analyzed by one-way ANOVA test. Data were shown in mean ± standard deviation. *P* < 0.05 was considered to be statistically significant.

## Supplementary information

**Additional file 1: Figure S1.** (a) Normalized UV-visible spectra and (b) hydrodynamic diameter of AgNPs in ALF after 0, 1, 3, 7 or 14 day-incubation at 37 °C. **Figure S2.** Cell viability of HepG2, L-02, NCTC-1469 and Hepa1–6 cells treated with AgNPs at 0, 2, 4, 10, 20 or 50 *μ*g/mL for 24 h, respectively. **Figure S3.** (a) Collection of AgNPs-treated HepG2 cells by centrifugation at 1000 rpm for 3 min. (b) Collection of soluble and insoluble components in AgNPs-treated HepG2 cells by centrifugation at 12,000 rpm for 20 min. **Figure S4.** (a) Cell viability of untreated and EA-treated HepG2 cells at 5, 10, 20, 50, 100 or 200 *μ*M for 24 h (*n* = 5). (b) Normalized intracellular GST activity of untreated and EA-treated HepG2 cells at 20, 50 or 100 *μ*M for 24 h (*n* = 3). (c) Intracellular ROS generation in untreated and EA-treated HepG2 cells at 10, 20, 50 or 100 *μ*M for 1 h or 3 h. Asterisk (*) denotes *P* < 0.05, compared to untreated cells. **Figure S5.** Hyperspectral images for untreated and AgNPs- or AuNPs-treated HepG2 cells at 10 *μ*g/mL for 24 h under different magnifications. (a) original magnification, × 10,000. (b) original magnification, × 20,000. **Figure S6.** Cell viability of HepG2 cells treated with PVP 8 k or PVP 40 k at 0, 4, 8, 10 or 50 *μ*g/mL for 24 h, respectively. **Table S1.** Composition (g L^− 1^) and pH of PBS and ALF solution. **Table S2.** Protein Identification by “protocol 1”. **Table S3.** Protein Identification by “protocol 2”.

## Data Availability

The datasets used and/or analyzed during the current study are available from the corresponding author on reasonable request.
