# Supplementary material for: Protein target identification and toxicological mechanism investigation of silver nanoparticles-induced hepatotoxicity by integrating proteomic and metallomic strategies
Source: Part Fibre Toxicol. 2019 Nov 27;16:46. doi: 10.1186/s12989-019-0322-4 (PMC6880521; doi:10.1186/s12989-019-0322-4)
Supplement: Supplementary file 1 — Additional file 1: Figure S1. (a) Normalized UV-visible spectra and (b) hydrodynamic diameter of AgNPs in ALF after 0, 1, 3, 7 or 14 day-incubation at 37 °C. Figure S2. Cell viability of HepG2, L-02, NCTC-1469 and Hepa1–6 cells treated with AgNPs at 0, 2, 4, 10, 20 or 50 μg/mL for 24 h, respectively. Figure S3. (a) Collection of AgNPs-treated HepG2 cells by centrifugation at 1000 rpm for 3 min. (b) Collection of soluble and insoluble components in AgNPs-treated HepG2 cells by centrifugation at 12,000 rpm for 20 min. Figure S4. (a) Cell viability of untreated and EA-treated HepG2 cells at 5, 10, 20, 50, 100 or 200 μM for 24 h (n = 5). (b) Normalized intracellular GST activity of untreated and EA-treated HepG2 cells at 20, 50 or 100 μM for 24 h (n = 3). (c) Intracellular ROS generation in untreated and EA-treated HepG2 cells at 10, 20, 50 or 100 μM for 1 h or 3 h. Asterisk (*) denotes P < 0.05, compared to untreated cells. Figure S5. Hyperspectral images for untreated and AgNPs- or AuNPs-treated HepG2 cells at 10 μg/mL for 24 h under different magnifications. (a) original magnification, × 10,000. (b) original magnification, × 20,000. Figure S6. Cell viability of HepG2 cells treated with PVP 8 k or PVP 40 k at 0, 4, 8, 10 or 50 μg/mL for 24 h, respectively. Table S1. Composition (g L− 1) and pH of PBS and ALF solution. Table S2. Protein Identification by “protocol 1”. Table S3. Protein Identification by “protocol 2”. [file 12989_2019_322_MOESM1_ESM.doc]

**Protein target identification and toxicological mechanism investigation of silver nanoparticles-induced hepatotoxicity by integrating proteomic and metallomic strategies**

Ming Xu^1,2,*^, Qiuyuan Yang^1^, Lining Xu^1^, Ziyu Rao^1^, Dong Cao^1^, Ming Gao^1^ and Sijin Liu^1,2^

^1^ State Key Laboratory of Environmental Chemistry and Ecotoxicology, Research Center for Eco-Environmental Sciences, Chinese Academy of Sciences, Beijing 100085, China

^2^ University of Chinese Academy of Sciences, Beijing 100049, China

* Correspondence: Ming Xu (mingxu@rcees.ac.cn)

Email address:

Qiuyuan Yang (yqy1908@163.com)

Lining Xu (18631719772@163.com)

Ziyu Rao (zyrao@rcees.ac.cn)

Dong Cao (dongcao@rcees.ac.cn)

Ming Gao (minggao@rcees.ac.cn)

Sijin Liu (sjliu@rcees.ac.cn)

| **Table S1** Composition (g L^-1^) and pH of PBS and ALF solution | | | |
| --- | --- | --- | --- |
| PBS | | ALF | |
| NaCl | 1.136 | NaCl | 3.210 |
| Na_2_HPO_4_ | 7.948 | NaOH | 6.000 |
| KH_2_PO_4_ | 0.272 | CaCl_2_ | 0.097 |
| KCl | 0.194 | NaH_2_PO_4_⋅7H2O | 0.179 |
|  |  | NaSO_4_ | 0.039 |
|  |  | MgCl_2_⋅6H2O | 0.106 |
|  |  | Glycerin | 0.059 |
|  |  | Citric acid | 20.800 |
|  |  | Sodium citrate dehydrate | 0.077 |
|  |  | Sodium tartrate dehydrate | 0.090 |
|  |  | Sodium lactate | 0.085 |
|  |  | Sodium pyruvate | 0.086 |
|  |  | Formaldehyde | 1.000 mL |
| pH | 7.2−7.4 | pH | 4.5−5.0 |

**Figure S1.** (a) Normalized UV-visible spectra and (b) hydrodynamic diameter of AgNPs in ALF after 0, 1, 3, 7 or 14 day-incubation at 37 °C.

**Figure S2.** Cell viability of HepG2, L-02, NCTC-1469 and Hepa1-6 cells treated with AgNPs at 0, 2, 4, 10, 20 or 50 *μ*g/mL for 24 h, respectively.

**Figure S3.** (a) Collection of AgNPs-treated HepG2 cells by centrifugation at 1,000 rpm for 3 min. (b) Collection of soluble and insoluble components in AgNPs-treated HepG2 cells by centrifugation at 12,000 rpm for 20 min.

| **Table S2.** **Protein Identification by “protocol 1”.** | | | | |
| --- | --- | --- | --- | --- |
| **No.** | **Protein Name** | **Accession No. *^a^*** | **Score *^b^*** | **Molecular Weight** |
| 1 | Myosin-9 | P35579 | 1271 | 227646 |
| 2 | U5 small nuclear ribonucleoprotein 200 kDa helicase | O75643 | 280 | 246006 |
| 3 | Eukaryotic translation initiation factor 4 gamma 1 | Q04637 | 29 | 226532 |
| 4 | Protein N-terminal glutamine amidohydrolase | Q96HA8 | 29 | 23680 |
| 5 | Caspase recruitment domain-containing protein 16 | Q5EG05 | 24 | 10737 |
| 6 | Beta-defensin 130 | Q30KQ2 | 22 | 8736 |
| 7 | Heterogeneous nuclear ribonucleoprotein U | Q00839 | 454 | 91269 |
| 8 | ATP-citrate synthase | P53396 | 120 | 121674 |
| 9 | Unconventional myosin-Ie | Q12965 | 40 | 127062 |
| 10 | KAT8 regulatory NSL complex subunit 1 | Q7Z3B3 | 30 | 121025 |
| 11 | Elongation factor 2 | P13639 | 1007 | 96246 |
| 12 | N-alpha-acetyltransferase 15 | Q9BXJ9 | 193 | 102462 |
| 13 | Zinc phosphodiesterase ELAC protein 2 | Q9BQ52 | 30 | 92219 |
| 14 | Keratin, type I cytoskeletal 10 | P13645 | 30 | 58827 |
| 15 | Nucleolin | P19338 | 27 | 76614 |
| 16 | Nucleolar RNA helicase 2 | Q9NR30 | 26 | 87344 |
| 17 | Protein LMBR1L | Q6UX01 | 21 | 55209 |
| 18 | Heat shock protein HSP 90-beta | P08238 | 1024 | 83554 |
| 19 | Heat shock protein HSP 90-alpha | P07900 | 461 | 85006 |
| 20 | Ribonucleoside-diphosphate reductase large subunit | P23921 | 892 | 90925 |
| 21 | Heat shock 70 kDa protein 1A | P0DMV8 | 419 | 70294 |
| 22 | Probable ATP-dependent RNA helicase DDX5 | P17844 | 40 | 69148 |
| 23 | Pyruvate kinase PKM | P14618 | 1625 | 58470 |
| 24 | Keratin, type I cytoskeletal 9 | P35527 | 469 | 62255 |
| 25 | Keratin, type II cytoskeletal 1 | P04264 | 675 | 66170 |
| 26 | Non-POU domain-containing octamer-binding protein | Q15233 | 403 | 54311 |
| 27 | T-complex protein 1 subunit theta | P50990 | 151 | 60153 |
| 28 | Tyrosine--tRNA ligase | P54577 | 106 | 59448 |
| 29 | T-complex protein 1 subunit alpha | P17987 | 102 | 60819 |
| 30 | UDP-glucose 6-dehydrogenase | O60701 | 97 | 55024 |
| 31 | Probable ATP-dependent RNA helicase DDX17 | Q92841 | 90 | 80272 |
| 32 | 60 kDa heat shock protein | P10809 | 59 | 61187 |
| 33 | Fascin | Q16658 | 424 | 55123 |
| 34 | Putative eukaryotic translation initiation factor 2 subunit 3-like protein | Q2VIR3 | 90 | 51766 |
| 35 | Tubulin beta-2B chain | Q9BVA1 | 36 | 49953 |
| 36 | Putative elongation factor 1-alpha-like 3 | Q5VTE0 | 1837 | 50495 |
| 37 | Eukaryotic initiation factor 4A-I | P60842 | 731 | 46353 |
| 38 | Eukaryotic initiation factor 4A-III | P38919 | 528 | 47126 |
| 39 | Elongation factor 1-gamma | P26641 | 280 | 50429 |
| 40 | 60S ribosomal protein L4 | P36578 | 276 | 47953 |
| 41 | Eukaryotic peptide chain release factor GTP-binding subunit ERF3B | Q8IYD1 | 137 | 68883 |
| 42 | 60S ribosomal protein L3 | P39023 | 133 | 46365 |
| 43 | 6-phosphogluconate dehydrogenase, decarboxylating | P52209 | 90 | 53619 |
| 44 | Keratin, type II cytoskeletal 8 | P05787 | 78 | 53704 |
| 45 | Actin | P60709 | 1028 | 42052 |
| 46 | Keratin, type I cytoskeletal 18 | P05783 | 633 | 48029 |
| 47 | Elongation factor Tu | P49411 | 128 | 49542 |
| 48 | Adenosylhomocysteinase | P23526 | 29 | 47716 |
| 49 | Sulfotransferase 1C3 | Q6IMI6 | 29 | 35889 |
| 50 | Elongation factor 1-alpha 2 | Q05639 | 28 | 50470 |
| 51 | Elongation factor 1-alpha 1 | P68104 | 24 | 50141 |
| 52 | Titin | Q8WZ42 | 25 | 3816030 |
| 53 | Glyceraldehyde-3-phosphate dehydrogenase | P04406 | 849 | 36201 |
| 54 | Nascent polypeptide-associated complex subunit alpha | E9PAV3 | 356 | 205422 |
| 55 | 60S acidic ribosomal protein P0-like | Q8NHW5 | 322 | 34514 |
| 56 | Annexin A1 | P04083 | 162 | 38918 |
| 57 | Malate dehydrogenase | P40925 | 54 | 36631 |
| 58 | L-lactate dehydrogenase B chain | P07195 | 40 | 36900 |
| 59 | Putative L-aspartate dehydrogenase | A6ND91 | 35 | 29946 |
| 60 | Annexin A2 | P07355 | 832 | 38808 |
| 61 | Aldose reductase | P15121 | 107 | 36230 |
| 62 | L-lactate dehydrogenase A chain | P00338 | 77 | 36950 |
| 63 | Deoxyribonuclease-1-like 1 | P49184 | 29 | 33893 |
| 64 | 40S ribosomal protein S3 | P23396 | 1134 | 26842 |
| 65 | Guanine nucleotide-binding protein subunit beta-2-like 1 | P63244 | 510 | 35511 |
| 66 | 40S ribosomal protein S2 | P15880 | 360 | 31590 |
| 67 | DNA replication licensing factor MCM5 | P33992 | 227 | 82286 |
| 68 | 40S ribosomal protein S6 | P62753 | 211 | 28834 |
| 69 | 40S ribosomal protein S3a | P61247 | 59 | 29945 |
| 70 | 60S ribosomal protein L13 | P26373 | 273 | 24304 |
| 71 | 60S ribosomal protein L8 | P62917 | 31 | 28025 |
| 72 | Heterogeneous nuclear ribonucleoprotein A1-like 2 | Q32P51 | 28 | 34225 |
| 73 | Glutathione S-transferase Mu 3 | P21266 | 98 | 26998 |
| 74 | 40S ribosomal protein S8 | P62241 | 97 | 24475 |
| 75 | Calcium/calmodulin-dependent protein kinase kinase 1 | Q8N5S9 | 26 | 55735 |
| 76 | Peroxiredoxin-1 | Q06830 | 307 | 22324 |
| 77 | 60S ribosomal protein L13a | P40429 | 126 | 23619 |
| 78 | Ras-related protein Rab-1B | Q9H0U4 | 115 | 22328 |
| 79 | 60S ribosomal protein L15 | P61313 | 115 | 24245 |
| 80 | Cytochrome P450 1B1 | Q16678 | 23 | 60846 |
| 81 | 40S ribosomal protein S7 | P62081 | 134 | 22113 |
| 82 | 40S ribosomal protein S9 | P46781 | 59 | 22635 |
| 83 | 60S ribosomal protein L23a | P62750 | 926 | 17684 |
| 84 | 60S ribosomal protein L11 | P62913 | 116 | 20468 |
| 85 | Eukaryotic translation initiation factor 1A | P47813 | 71 | 16564 |
| 86 | 60S ribosomal protein L26 | P61254 | 48 | 17258 |
| 87 | ADP-ribosylation factor 4 | P18085 | 46 | 20511 |
| 88 | 40S ribosomal protein S11 | P62280 | 30 | 18431 |
| 89 | Ubiquitin-associated protein 2-like | Q14157 | 30 | 114535 |
| *^a^* UniProt. *^b^* Mascot score. | | | | |

| **Table S3.** **Protein Identification by “protocol 2”.** | | | | |
| --- | --- | --- | --- | --- |
| **No.** | **Protein Name** | **Accession No. ^a^** | **Score ^b^** | **Molecular Weight** |
| 1 | Peptidylprolyl isomerase A | P62937 | 219 | 18012 |
| 2 | 60S ribosomal protein L7 | P18124 | 127 | 29226 |
| 3 | Triosephosphate isomerase isoform 1 | P60174 | 122 | 30791 |
| 4 | 40S ribosomal protein S4, X isoform | P62701 | 88 | 29598 |
| 5 | Peroxiredoxin-6 | P30041 | 85 | 25035 |
| 6 | 60S ribosomal protein L24 | P83731 | 77 | 17779 |
| 7 | 50S ribosomal protein L11 | A0A2P9ASX5 | 76 | 15194 |
| 8 | Transgelin 2 | P37802 | 73 | 22391 |
| 9 | Histone H1.2 | P16403 | 65 | 21365 |
| 10 | Ribosomal protein S19 binding protein 1, isoform CRA_a | A0A024R1T1 | 63 | 15434 |
| 11 | 60S Ribosomal Protein L12 LIKE protein | Q76P68 | 62 | 17926 |
| 12 | Stathmin | P16949 | 59 | 17303 |
| 13 | Galectin-3 | P17931 | 56 | 26152 |
| 14 | Peroxiredoxin-1 | Q06830 | 55 | 22110 |
| 15 | Protein/nucleic acid deglycase DJ-1 | Q99497 | 55 | 19891 |
| 16 | Proteasome subunit alpha type-1 | P25786 | 53 | 29556 |
| 17 | Heat shock protein beta-1 | P04792 | 45 | 22783 |
| 18 | 60S ribosomal protein L8 | P62917 | 38 | 28025 |
| 19 | 60S ribosomal protein L29 | P47914 | 41 | 17752 |
| 20 | Elongation factor 1-beta | P24534 | 38 | 24764 |
| 21 | Myosin regulatory light chain 12A | P19105 | 38 | 19794 |
| 22 | Nucleoside diphosphate kinase A | P15531 | 36 | 17149 |
| 23 | 60S ribosomal protein L10a | P62906 | 36 | 24831 |
| 24 | Clathrin light chain A | P09496 | 33 | 27077 |
| 25 | Adenylate kinase 2, mitochondrial | P54819 | 33 | 26478 |
| 26 | Electron transfer flavoprotein subunit beta | P38117 | 32 | 27844 |
| 27 | 60S ribosomal protein L21 | P46778 | 30 | 18565 |
| 28 | 60S ribosomal protein L7a | Q5T8U3 | 29 | 21545 |
| 29 | Cofilin-1 | P23528 | 28 | 18502 |
| 30 | ANP32A protein | Q6PKH8 | 26 | 24068 |
| 31 | Phosphoglycerate mutase 2 | P15259 | 26 | 28766 |
| 32 | Glutathione S-transferase P | P09211 | 26 | 23356 |
| 33 | Platelet-activating factor acetylhydrolase IB subunit gamma | Q15102 | 24 | 25734 |
| 34 | 60S ribosomal protein L18 | Q07020 | 23 | 21634 |
| 35 | Platelet-activating factor acetylhydrolase IB subunit beta | P68402 | 22 | 25569 |
| 36 | Myosin light chain 6B | P14649 | 20 | 22764 |
| *^a^* UniProt. *^b^* Mascot score. | | | | |

**Figure S4.** (a) Cell viability of untreated and EA-treated HepG2 cells at 5, 10, 20, 50, 100 or 200 *μ*M for 24 h (n = 5). (b) Normalized intracellular GST activity of untreated and EA-treated HepG2 cells at 20, 50 or 100 *μ*M for 24 h (n = 3). (c) Intracellular ROS generation in untreated and EA-treated HepG2 cells at 5, 10, 20, 50, 100 or 200 *μ*M for 1 h or 3 h. Asterisk (*) denotes *P* < 0.05, compared to untreated cells.

**Figure S5.** Hyperspectral images for untreated and AgNPs- or AuNPs-treated HepG2 cells at 10 *μ*g/mL for 24 h under different magnifications. (a) original magnification, × 10,000. (b) original magnification, × 20,000.

**Figure S6.** Cell viability of HepG2 cells treated with PVP 8k or PVP 40k at 0, 4, 8, 10 or 50 *μ*g/mL for 24 h, respectively.
